# Acquired Drug Resistance in *Mycobacterium tuberculosis* and Poor Outcomes among Patients with Multidrug-Resistant Tuberculosis

**DOI:** 10.3201/eid2106.141873

**Published:** 2015-06

**Authors:** Russell R. Kempker, Maia Kipiani, Veriko Mirtskhulava, Nestani Tukvadze, Matthew J. Magee, Henry M. Blumberg

**Affiliations:** Emory University School of Medicine, Atlanta, Georgia, USA (R.R. Kempker, H.M. Blumberg);; National Center for Tuberculosis and Lung Diseases, Tbilisi, Georgia (M. Kipiani, N. Tukvadze);; Davit Tvildiani Medical University, Tbilisi (V. Mirtskhulava);; Georgia State University School of Public Health, Atlanta (M.J. Magee)

**Keywords:** tuberculosis and other mycobacteria, Mycobacterium tuberculosis, bacteria, multidrug-resistant tuberculosis, MDR TB, extensively drug-resistant tuberculosis, XDR TB, antimicrobial resistance, multidrug resistance, acquired drug resistance, treatment outcomes

## Abstract

Rates and risk factors for acquired drug resistance and association with outcomes among patients with multidrug-resistant tuberculosis (MDR TB) are not well defined. In an MDR TB cohort from the country of Georgia, drug susceptibility testing for second-line drugs (SLDs) was performed at baseline and every third month. Acquired resistance was defined as any SLD whose status changed from susceptible at baseline to resistant at follow-up. Among 141 patients, acquired resistance in *Mycobacterium tuberculosis* was observed in 19 (14%); prevalence was 9.1% for ofloxacin and 9.8% for capreomycin or kanamycin. Baseline cavitary disease and resistance to >6 drugs were associated with acquired resistance. Patients with *M. tuberculosis* that had acquired resistance were at significantly increased risk for poor treatment outcome compared with patients without these isolates (89% vs. 36%; p<0.01). Acquired resistance occurs commonly among patients with MDR TB and impedes successful treatment outcomes.

## Abstract 2

Acquired drug resistance is common and an impediment to successful treatment outcomes.

The World Health Organization (WHO) reported that control efforts are off-track in managing multidrug-resistant tuberculosis (MDR TB) and that addressing this problem is a priority (*1*). In 2013, WHO estimated that there were 480,000 new cases of MDR TB and 210,000 MDR TB–related deaths (*2*). MDR TB, defined as resistance to first-line drugs isoniazid and rifampin, has been associated with worse treatment outcomes than for drug-susceptible TB (*3*). A primary reason for worse treatment outcomes for MDR TB is use of second-line drugs (SLDs), which are costly, poorly tolerated, and suboptimally effective and require a prolonged treatment duration.

SLD treatment for MDR TB might also increase risk for further acquired drug resistance. Acquired resistance among *Mycobacterium tuberculosis* isolates from MDR TB patients is a concern because this resistance would leave clinicians with few effective drugs and might lead to development of extensively drug-resistant (XDR) TB, which is defined as resistance to a fluoroquinolone and >1 injectable drug (amikacin, kanamycin, or capreomycin) (*4*), in addition to isoniazid and rifampin. XDR TB has been associated with treatment outcomes much worse than outcomes for MDR TB (*5*).

A case of drug-resistant TB occurs by primary transmission of drug-resistant *M*. *tuberculosis* strains or acquired resistance during TB treatment. For acquired resistance, *M. tuberculosis* is believed to develop resistance by spontaneous chromosomal mutations (*6*). Given that frequencies of *M. tuberculosis* mutations that correlate with drug resistance occur infrequently and resistance mutations for different drugs are believed to be unlinked, additional drug resistance is unlikely when ≥3 effective drugs are used in combination (*6*). For inadequate drug treatment caused by poor regimen selection, inadequate drug supply, nonadherence, or subtherapeutic drug concentrations, subpopulations of drug-resistant *M. tuberculosis* might be selected for, amplified, and become the predominant strain. Limited data suggest that the risk for acquired resistance is higher among persons with MDR TB than drug-susceptible TB (*7*–*10*). Data for risk factors for acquired resistance among MDR TB patients during treatment and their effect on outcomes are limited to a few studies (*8*,*11*,*12*).

On the basis of prior results from our group, we hypothesized that cavitary disease would increase the risk for acquired resistance (*13*). Because infection with *M. tuberculosis* strains with increasing drug resistance is associated with worse patient outcomes, we also hypothesized that acquired resistance would be associated with a poor outcome (*5*).

To address research questions generated by these hypotheses, we studied a cohort of MDR TB patients in Georgia, 1 of 27 countries with a high incidence of MDR TB, as designated by WHO (*1*). In 2012, 9% of newly diagnosed cases and 31% of re-treatment TB cases in Georgia were MDR TB (*1*). In 2008, with support from the Global Fund (http://www.theglobalfund.org/en/?gclid=CO2244zBqsQCFdgNgQodQAkANA) and the Green Light Committee (GLC) (http://www.who.int/tb/challenges/mdr/greenlightcommittee/en/), Georgia was the first low-to-middle income country to achieve universal access to diagnosis and treatment of MDR TB. However, despite availability of culture and molecular diagnostic methods and use of recommended SLD regimens, MDR TB treatment outcomes have remained suboptimal compared with other similar settings (*14*). By assessing prevalence of acquired resistance and its effect on treatment outcomes, we aimed to obtain data that might lead to improved management of MDR TB patients in Georgia and other countries that have drug-resistant TB.

## Methods

### Study Population

We conducted a retrospective study of patients with pulmonary MDR TB treated through the National Center for Tuberculosis and Lung Diseases (NCTLD) in Tbilisi, Georgia. All patients were sputum smear–positive for acid-fast bacilli (AFB) and culture-positive for *M. tuberculosis* at baseline and had MDR TB confirmed by conventional drug susceptibility testing (DST). Patients with MDR TB were given a diagnosis during March 2009–October 2012, as described in a study that evaluated the clinical effect of a rapid diagnostic test for detection of MDR TB (*15*). Approval for this study was obtained from the Georgia NCTLD and Emory University (Atlanta, GA, USA) Institutional Review Boards.

### Cultures and Drug Susceptibility Testing

Direct sputum smears with Ziehl-Neelsen staining were examined by light microscopy at a sputum microscopy center, and 1 sputum smear sample positive for acid-fast bacilli (AFB) was sent to the National Reference Laboratory (NRL) at NCTLD, where it was processed as reported (*16*). Cultures were prepared by using Löwenstein-Jensen–based solid medium or the MGIT 960 broth culture system (Becton Dickinson, Franklin Lakes, NJ, USA). Cultures with positive results by either method were confirmed to be *M. tuberculosis* complex by using the MTBDR*plus* assay (Hain Lifescience, Nehren, Germany) and the Capilia TB assay (Tauns Laboratories, Inc., Shizuoka, Japan) (*16*).

DST for first-line drugs was performed as described (*16*). DST for SLDs was performed by using the proportion method and Löwenstein-Jensen medium with the following drug concentrations: ethionamide, 40.0 μg/mL; ofloxacin, 2.0 μg/mL; p-aminosalicylic acid, 0.5 μg/mL; capreomycin, 40.0 μg/mL; and kanamycin, 30.0 μg/mL (*17*). The NRL has undergone external quality assessment by the WHO Supranational TB Reference Laboratory (Antwerp, Belgium) annually since 2005. In 2012, a certificate from the WHO Supranational TB Reference Laboratory was received by the NRL for successfully passing DST quality assurance testing for isoniazid, rifampin, ethambutol, kanamycin, capreomycin, and ofloxacin.

As per standard of care, follow-up sputum smears and cultures were obtained monthly during the intensive phase of MDR TB treatment (minimum 6 months). Second-line DST was performed at baseline and was recommended at 3 and 6 months if culture results remained positive. During the continuation phase, sputum smears and cultures were obtained every 3 months, and second-line DST was recommended for all positive cultures.

### Data Collection

A standardized data form was used to abstract data from medical charts, patient treatment cards, the NCTLD surveillance database, and the NRL database. Information was collected about sociodemographic characteristics, TB history, signs and symptoms, treatment regimens, outcomes; and all sputum smear, culture, and DST results. Data were entered into a REDCap database (*18*).

### Definitions

Acquired resistance was defined as any SLD that was susceptible on baseline DST and resistant on any subsequent DST result. Time to MDR TB treatment initiation was defined as time from initial diagnostic sputum collection to start of SLD therapy. Initial MDR TB treatment was defined as any drug received <30 days of starting an SLD regimen. Treatment interruption was defined as a continuous interruption for >1 SLDs for >1 week. Final treatment outcomes were determined using WHO criteria (*19*). A favorable outcome was defined as cure or treatment completion; a poor outcome was defined as treatment failure, death during treatment, or loss to follow up (LFU) (formerly known as default). Two alternative classifications were used in secondary analyses: 1) patients with a negative culture result at time of LFU were included as a favorable outcome, and 2) patients with a poor outcome were defined as treatment failure or death. LFU patients were excluded from analysis.

### Treatment

The NCTLD Drug Resistance TB Treatment Committee provided initial guidance on choosing an empiric SLD regimen for patients initiating MDR TB treatment. After second-line DST results were available, treatment regimens were individualized if needed and guided by WHO recommendations. When possible, regimens were designed to include at least 4 drugs to which an *M. tuberculosis* isolate was susceptible. All treatment regimens included a fluoroquinolone, pyrazinamide, and capreomycin or kanamycin for >6 months. All patients received directly observed therapy. Patients initiated therapy as inpatients before transitioning to outpatient treatment. Patients were recommended to remain hospitalized until showing sputum smear or culture conversion and clinical improvement.

### Data Analysis

Data were analyzed by using SAS software version 9.3 (SAS Institute, Cary, NC, USA). For descriptive statistics, differences in categorical variables were tested by using the χ^2^ or Fisher exact tests. The Wilcoxon-Mann-Whitney test was used for comparing non–normally distributed continuous variables. A 2-sided p value <0.05 was considered significant. A logistic regression model was used to estimate the independent association of potential risk factors with acquired resistance and the adjusted association of acquired resistance with a poor outcome. Logistic model building and covariate selection was based on purposeful selection of patient-level factors as described (*20*). Additional logistic regression models using our alternative definitions of poor outcome as defined above were used.

## Results

### Study Cohort

**Figure 1:**
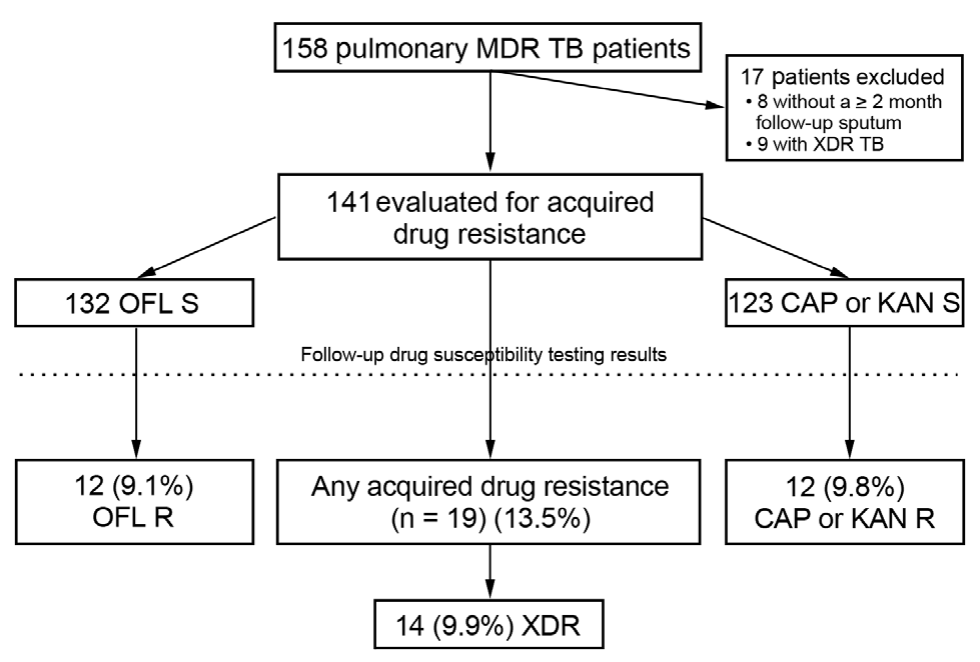
Cohort diagram of patients with multidrug-resistant tuberculosis (MDR TB) depicting rates of acquired drug resistance, Georgia, March 2009–October 2012. XDR TB, extensively drug-resistant tuberculosis; OFL, ofloxacin; S, susceptible; CAP, capreomycin; KAN, kanamycin; R, resistant.

A total of 158 patients with pulmonary MDR TB were included in the study. For analysis of acquired resistance, 17 patients were excluded because of XDR TB at baseline or absence of a 2-month or later follow-up sputum examination (Figure 1). The remaining 141 patients had a mean age of 37.9 years; most (73%) were men. Less than half (44%) had a history of TB treatment. *M. tuberculosis* isolates with baseline resistance to capreomycin or kanamycin were found in 33% of patients, and isolates with baseline resistance to ofloxacin were found in 6%. Other patient characteristics are shown in Table 1.

**Table 1:** Characteristics of patients with multidrug-resistant tuberculosis stratified by acquired resistance to second-line drugs, Georgia, March 2009–October 2012*

Characteristic	Overall, n = 141	Acquired resistance to second-line drugs		p value†
		Yes, n = 19	No, n = 122	
Median age, y (IQR)	34.9 (27–46)	41.4 (30–53)	37.3 (27–45)	0.28
Male sex	103 (73)	16 (84)	87 (71)	0.24
Married	73 (52)	10 (53)	63 (52)	0.94
Employed	19 (14)	1 (5)	18 (15)	0.26
History of imprisonment	40 (28)	6 (32)	34 (28)	0.74
Diabetes mellitus	16 (11)	1 (5)	15 (12)	0.37
Hepatitis C	16 (11)	3 (16)	13 (11)	0.51
HIV positive	6 (4)	1 (5)	5 (4)	0.82
Median BMI, kg/m^2^ (IQR)	20.5 (2.7)	20.0 (17.5–21.1)	20.2 (18.9–22.5)	0.049
BMI ≤18.5 kg/m^2^	35 (25)	7 (37)	28 (23)	0.19
History of TB	62 (44)	10 (53)	52 (43)	0.41
Prior TB treatment				0.27
None	79 (56)	9 (47)	70 (57)	NA
First-line	52 (37)	7 (37)	45 (37)	NA
Second-line	10 (7)	3 (16)	7 (6)	NA
Baseline cavitary disease	30 (21)	11 (58)	19 (16)	<0.01
Median no. drugs to which baseline isolate was resistant (IQR)	5 (5–6)	6 (5–7)	5 (5–6)]	0.02
Resistant to ≥6 drugs on baseline DST	60 (43)	14 (74)	46 (38)	<0.01
Baseline drug resistance category				
MDR only	85 (60)	8 (42)	77 (63)	0.01
MDR + ofloxacin resistant	9 (6)	4 (21)	5 (4)	NA
MDR + capreomycin or kananmycin resistant	47 (33)	7 (37)	40 (33)	NA
Initial treatment inpatient	45 (32)	9 (47)	36 (30)	0.12
Starting SLDs >30 days after TB diagnosis	66 (47)	6 (32)	60 (49)	0.15
Median known active drugs in initial regimen (IQR)‡	3 (2–3)	2 (1–3)	3 (2–4)	0.05
Initial MDR TB treatment				
Pyrazinamide	139 (99)	19 (100)	120 (98)	0.57
Prothionamide	141 (100)	19 (100)	122 (100)	1.00
Capreomycin	65 (46)	13 (68)	52 (43)	0.04
Kanamycin	82 (58)	6 (32)	76 (62)	0.01
Levofloxacin	134 (95)	18 (94)	116 (95)	0.95
Cycloserine	135 (96)	19 (100)	116 (95)	0.32
p-aminosalicyclic acid	140 (99)	19 (100)	121 (99)	0.69
Treatment interruption	57 (40)	12 (63)	45 (37)	0.03
Baseline AFB sputum smear value >3+	46 (33)	12 (63)	34 (28)	<0.01
Sputum culture positive, mo§				
2	121 (86)	19 (100)	102 (84)	0.06
4	80 (57)	17 (90)	63 (52)	<0.01
6	48 (34)	16 (84)	32 (26)	<0.01
Sputum smear positive, mo§				
2	115 (82)	19 (100)	96 (79)	0.03
4	77 (55)	17 (90)	60 (49)	<0.01
6	39 (28)	15 (79)	24 (20)	<0.01

*Values are no. (%) unless otherwise indicated. IQR, interquartile range; BMI, body mass index; TB, tuberculosis; NA, not applicable; DST, drug susceptibility testing; MDR, multidrug resistant; SLDs, second-line drugs; AFB, acid-fast bacilli. †Comparing persons with and without acquired resistance by using χ^2^ or Fischer exact tests for categorical variables and Wilcoxon-Mann-Whitney test for continuous variables. ‡DST was performed for streptomycin, isoniazid, rifampin, ethambutol, ofloxacin, ethionamide, kanamycin, capreomycin, and p-aminosalicyclic acid. §Time from initiation of SLD treatment for MDR TB.

### Acquired Resistance

Of the 141 patients evaluated for acquired resistance, 32 patients had ≥1 follow-up DST performed, including 40% of patients with a positive 4-month culture and 52% with a positive 6-month culture. A total of 22 patients had different follow-up DST results that showed a change in resistance pattern, including 19 (13.5%) with acquired resistance and 3 (2.1%) with follow-up DST showing a reversion to susceptibility for 1 SLD. Median time to initial development of acquired resistance was 142 days (range 85–480 days), including 200 days for capreomycin or kanamycin and 149 days for ofloxacin. Of 132 patients infected with *M. tuberculosis* that had baseline ofloxacin susceptibility, ofloxacin resistance developed in isolates from 12 (9.1%) patients. Among 123 patients infected with isolates that had baseline susceptibility to capreomycin, kanamycin, or both, resistance to capreomycin or kanamycin developed in 12 (9.8%) (Figure 1). Of 12 patients infected with isolates that had acquired resistance to an injectable drug, resistance to capreomycin and kanamycin developed in isolates from 5 patients, resistance to capreomycin developed in isolates with baseline kanamycin resistance from 3 patients, and susceptibility to capreomycin or kanamycin remained in isolates from 4 patients.

Among 19 patients infected with isolates that had acquired resistance, increasing resistance to 1 drug developed in isolates from 9 patients, to 2 drugs in isolates from 8 patients, and to 3 and 4 additional drugs in isolates from 1 patient each. Almost all acquired resistance was to ofloxacin, capreomycin, or kanamycin; 2 patients each had isolates with acquired resistance to ethionamide and p-aminosalicylic acid. Acquired resistance led to emergence of XDR TB in 14 (74%) of 19 patients.

Patients with and without isolates that had acquired resistance were similar in regards to age, sex, prior TB, and other characteristics (Table 1). In contrast, patients who had isolates with acquired resistance had a lower mean baseline body mass index (19.1 vs. 20.7 kg/m^2^; p = 0.02) and a higher prevalence of baseline cavitary disease (58% vs. 16%; p<0.01) and were more likely to have isolates resistant to >6 drugs at baseline DST (74% vs. 38%; p = 0.01) than patients who had isolates without acquired resistance. Patients with isolates that had acquired resistance were less likely to receive kanamycin (32% vs. 62%; p = 0.01) as part of their initial MDR TB treatment regimen. Regarding sputum examinations, patients with isolates that had acquired resistance were more likely to have baseline AFB sputum smear values >3+ (63% vs. 28%; p<0.01) and to be sputum smear and culture positive at 4 and 6 months (Table 1) than patients without isolates that had acquired resistance.

Factors associated with acquired resistance by univariate analysis were baseline cavitary disease, high baseline drug resistance, baseline ofloxacin resistance, number of known active drugs in the initial MDR TB treatment regimen, initial AFB sputum smear result >3+, and sputum smear or culture positivity at 4 and 6 months (Table 2). By multivariate analysis, factors associated with acquired resistance were baseline cavitary disease (adjusted odds ratio [aOR] 5.21, 95% CI 1.56–17.38); resistance to >6 drugs at baseline DST (aOR 5.31, 95% CI 1.50–18.77); and sputum smear positivity at 4 months (aOR 6.54, 95% CI 1.23–34.88).

**Table 2:** Risk factors for acquired resistance to second-line drugs among patients treated for multidrug-resistant tuberculosis, Georgia, March 2009–October 2012*

Risk factor	Univariate analysis, OR (95% CI)	p value	Multivariate analysis, aOR (95% CI)	p value
Baseline characteristic				
Median age >35 y	1.96 (0.72–5.30)	0.19	–	–
Male sex	2.15 (0.59–7.83)	0.25	–	–
BMI ≤18.5 kg/m^2^	1.96 (0.70–5.45)	0.20	3.73 (0.98–14.14)	0.053
History of TB	1.50 (0.57–3.94)	0.42	–	–
Prior receipt of second-line TB drugs	3.08 (0.72–13.13)	0.13	–	–
Diabetes	0.40 (0.05–3.19)	0.38	–	–
Hepatitis C	1.57 (0.40–6.13)	0.52	–	–
HIV	1.30 (0.14–11.78)	0.82	–	–
Cavitary disease	7.45 (2.65–20.96)	<0.01	5.21 (1.56–17.38)	<0.01
No. of drugs to which baseline isolate was resistant/drug (IQR)	1.63 (1.05–2.51)	0.03	–	–
Resistant to ≥6 drugs by baseline DST	4.63 (1.56–13.68)	<0.01	5.31 (1.50–18.77)	0.01
Baseline ofloxacin resistant	6.24 (1.51–25.83)	0.01	–	–
Baseline capreomycin or kanamycin resistant	1.20 (0.44–3.27)	0.73	–	–
Known active drugs in initial regimen per drug	0.58 (0.35–0.99)	0.045	–	–
Follow-up characteristic				
Initial MDR TB treatment				
Capreomycin	2.92 (1.04–8.18)	0.04	–	–
Treatment interruption	2.93 (1.08–7.99)	0.04	–	–
>30 d to start SLDs	0.48 (0.17–1.34)	0.16	–	–
Baseline AFB sputum smear value >3+	4.44 (1.61–12.22)	<0.01	2.21 (0.66–7.48)	0.20
Sputum smear positive, mo†				
4	8.78 (1.95–39.66)	<0.01	6.54 (1.23–34.88)	0.03
6‡	15.31 (4.66–50.32)	<0.01	–	–

*OR, odds ratio; aOR, adjusted OR; –, not included in multivariate analysis; BMI, body mass index; TB, tuberculosis; IQR, interquartile range; DST, drug susceptibility testing; MDR, multidrug resistant; SLDs, second-line drugs; AFB, acid-fast bacilli. †Time from initiation of second-line drug treatment for MDR TB. ‡Significant by an alternative multivariate analysis model when replacing the variable sputum smear positive at 4 mo.

### Risk Factors for Poor Outcomes

**Figure 2:**
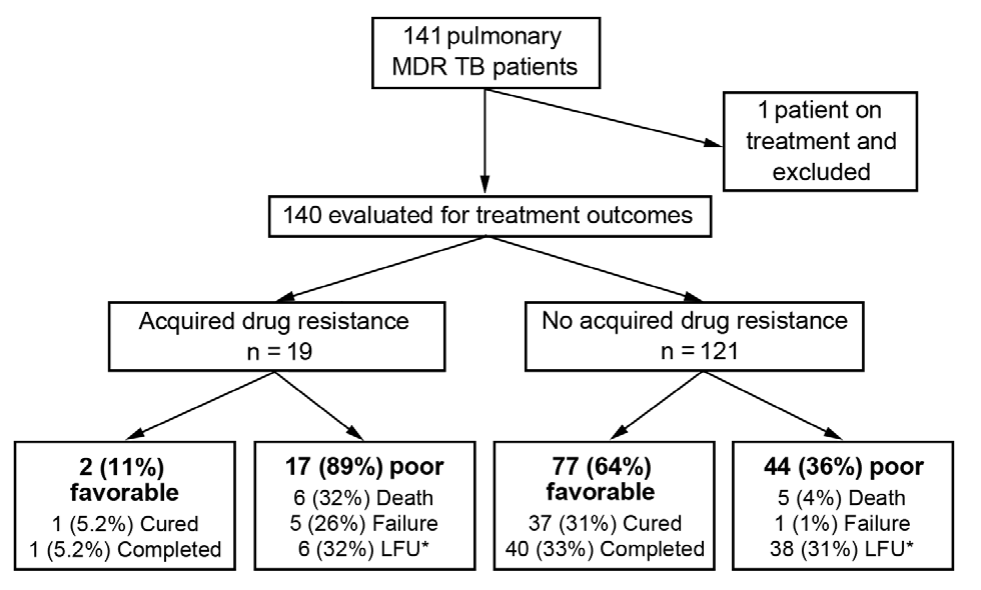
Final treatment outcomes for patients with multidrug-resistant tuberculosis (MDR TB), by acquired drug resistance status, Georgia, March 2009–October 2012. LFU, loss to follow up. *15 of 44 patients were culture positive at time of LFU, including all 6 patients with acquired resistance.

A total of 140 patients were evaluated for final treatment outcomes; 1 patient was excluded because he was still receiving treatment (Figure 2). Of the remaining 139 patients, 61 (44%) had a poor outcome. Poor outcomes were more frequent among patients with isolates that had acquired resistance than patients without these isolates (89% vs. 36%; p<0.01). Of 2 patients who had favorable outcomes and isolates that had acquired resistance, 1 underwent adjunctive surgery and 1 had an isolate with acquired resistance to ethionamide; these isolates remained susceptible to ofloxacin, kanamycin, and capreomycin. Most (44 of 61) poor outcomes were caused by LFU, and the remaining poor outcomes were caused by deaths (11) and treatment failure (6). Of LFU patients, 15 (34%) of 44 had positive sputum cultures at the time of LFU, including 6 with isolates that had acquired resistance. The 29 patients with negative cultures at the time of LFU received a median of 111 days of treatment for MDR TB (range 42–325 days), and the 15 patients with positive cultures received a median of 91 days of treatment (range 80–360 days).

Patients with a poor outcome were significantly more likely to have isolates with acquired resistance (28% vs. 3%; p<0.01) and be sputum smear positive at 4 and 6 months and sputum culture positive at 2, 4, and 6 months (Table 3) than patients with a favorable outcome. There were no other differences between groups (Table 3).

**Table 3:** Characteristics of patients with multidrug-resistant tuberculosis, by treatment outcome, Georgia, March 2009–October 2012*

Characteristic	Poor outcome, n = 61	Favorable outcome, n = 79	p value†
Acquired resistance to any second-line drug	17 (28)	2 (3)	<0.01
Median age, y	39.7	33.7	0.21
Male sex	49 (80)	53 (67)	0.08
History of imprisonment	21 (34)	19 (24)	0.18
Diabetes mellitus	6 (10)	10 (13)	0.60
Hepatitis C	9 (15)	7 (9)	0.28
HIV	3 (5)	3 (4)	0.75
BMI ≤18.5 kg/m^2^	14 (23)	21 (27)	0.63
History of TB	29 (48)	33 (42)	0.50
Prior TB treatment			0.77
None	32 (53)	46 (58)	NA
First-line	24 (39)	28 (35)	NA
Second-line	5 (8)	5 (6)	NA
Baseline cavitary disease	17 (28)	13 (17)	0.11
Median no. drugs to which baseline isolate was resistant	6	5	0.18
Resistant to ≥6 drugs on baseline DST	32 (53)	28 (35)	0.04
Baseline ofloxacin resistant	6 (10)	3 (4)	0.15
Baseline capreomycin kanamycin resistant	24 (39)	23 (29)	0.20
Starting SLDs >30 days	28 (46)	38 (48)	0.80
Initial MDR TB treatment regimen included			
Capreomycin	25 (41)	40 (51)	0.26
Kanamycin	35 (57)	46 (58)	0.92
Ever received			
Moxifloxacin	11 (18)	9 (12)	0.27
Clarithromycin	3 (5)	2 (3)	0.45
Augmentin	3 (5)	2 (3)	0.45
Clofazimine	3 (5)	3 (4)	0.75
Treatment interruption	29 (48)	27 (34)	0.11
Adjunctive surgery performed	3 (5)	4 (5)	0.97
Baseline sputum AFB smear value *>*3+	23 (38)	23 (29)	0.28
Sputum culture positive, mo‡			
2	58 (95)	62 (79)	<0.01
4	44 (72)	36 (46)	<0.01
6	34 (56)	14 (18)	<0.01
Sputum smear positive, mo‡			
2	53 (87)	61 (77)	0.14
4	42 (69)	35 (44)	<0.01
6	28 (46)	11 (14)	<0.01

*BMI, body mass index; TB, tuberculosis; NA, not applicable; DST, drug susceptibility testing; SLDs, second-line drugs; MDR, multidrug resistant; AFB, acid-fast bacilli. †Comparing persons with and without acquired resistance by using χ^2^ or Fischer exact tests for categorical variables and *t*-test or median test for continuous variables. ‡Time from initiation of second-line drug treatment for MDR TB.

Risk factors associated with a poor outcome by univariate analysis were acquired resistance, high baseline drug resistance, and sputum smear or culture positivity at 4 and 6 months (Table 4). Multivariate analysis showed that acquired resistance (aOR 8.05, 95% CI 1.56–41.66) and sputum smear positivity at 6 months (aOR 3.43, 95% CI 1.36–8.63) remained associated with a poor outcome. In our first alternative multivariate model, when we classified patients with a negative culture at time of LFU as a favorable outcome, acquired resistance was associated with a poor outcome (aOR 24.91, 95% CI 4.21–147.48). In a second alternative multivariate model, in which poor outcome was defined as treatment failure or death and LFU patients were excluded, acquired resistance remained associated with a poor outcome (aOR 38.44, 95% CI 5.96–247.73).

**Table 4:** Risk factors for poor treatment outcomes among patients treated for multidrug-resistant tuberculosis, Georgia, March 2009–October 2012*

Risk factor	Univariate analysis, OR (95% CI)	p value	Multivariate analysis, aOR (95% CI)	p value
Acquired resistance to any second-line drug	14.88 (3.28–67.42)	<0.01	8.05 (1.56–41.66)	0.01
Baseline characteristics				
Increasing age per year	1.02 (0.99–1.05)	0.14	1.02 (0.99–1.05)	0.26
Male sex	2.00 (0.91–4.40)	0.08	–	
BMI ≤18.5 kg/m^2^	0.82 (0.38–1.79)	0.62	–	–
History of TB	1.26 (0.65–2.48)	0.50	–	–
Prior receipt of second-line TB drugs	1.32 (0.37–4.79)	0.67	–	-
Diabetes mellitus	0.75 (0.26–2.20)	0.60	–	–
Hepatitis C	1.78 (0.62–509)	0.28	–	–
HIV	1.31 (0.26–6.73)	0.75	–	–
Baseline cavitary disease	1.96 (0.87–4.44)	0.11	0.72 (0.25–2.05)	0.54
Resistant to ≥6 drugs by baseline DST	2.01 (1.02–3.98)	0.04	1.45 (0.68–3.11)	0.34
No. drugs to which baseline isolate was resistant (IQR)	1.20 (0.90–1.59)	0.21	–	–
Drug resistance category				
Baseline ofloxacin resistant	2.76 (0.66–11.53)	0.16	–	–
Baseline capreomycin or kanamycin resistant	1.58 (0.78–3.20)	0.21	–	–
Follow-up characteristics				
Treatment interruption	1.75 (0.88–3.46)	0.11	–	–
>30 d to start SLDs	0.92 (0.47–1.79)	0.80	–	–
Initial capreomycin treatment	0.68 (0.35–1.33)	0.26	–	–
Baseline sputum smear AFB value >3+	1.47 (0.73–3.00)	0.28	–	–
Sputum smear positive, mo†				
4‡	2.78 (1.38–5.60)	<0.01	–	–
6	5.25 (2.33–11.81)	<0.01	3.43 (1.36–8.63)	0.01
Sputum culture positivity,mo				
4‡	3.09 (1.51–6.31)	<0.01	–	–
6‡	5.85 (2.71–12.59)	<0.01	–	–

*OR, odds ratio; aOR, adjusted OR; –, not included in multivariate analysis; BMI, body mass index; TB, tuberculosis; IQR, interquartile range; DST, drug susceptibility testing; SLDs, second-line drugs; AFB, acid-fast bacilli. †Time from initiation of second-line drug treatment for MDR TB. ‡Significant by alternative multivariate analysis models when replacing the variable sputum smear positive at 6 mo.

## Discussion

In a country with high rates of MDR TB, we found an alarmingly high rate of acquired drug resistance during SLD treatment (13.5%), including development of XDR TB (9.9%) and a strong association between acquired resistance and poor treatment outcomes (aOR 8.05, 95% CI 1.56–41.66). These high rates of acquired resistance were observed even though Georgia is a GLC-approved country, thus receiving quality-ensured SLDs and providing all treatment by directly observed therapy. In our study, baseline cavitary disease, high-grade smear positivity, increased drug resistance, and persistent smear positivity at follow-up sputum examinations were associated with acquired resistance. Although these risk factors might assist physicians in identifying those patients at increased risk for acquired resistance (and consequently poor outcomes), further evaluation is needed in evaluating optimal methods to treat patients who have isolates with acquired resistance.

Information on rates of acquired resistance among patients receiving second-line treatment is limited. A retrospective study of 536 MDR TB patients in western Siberia, Russia, found that XDR TB developed in 34 (6.4%); no information was provided on acquired resistance to quinolones or injectable drugs (*12*). Another study from the autonomous region of Abkhazia found that in a subpopulation of 47 MDR TB patients, XDR TB developed in 5 (11%) (*21*).

In the recently published Preserving Effective TB Treatment Study (PETTS), 832 MDR TB patients from 9 countries were prospectively followed up for acquired resistance (*11*). In that study, in comparison with our results, the rate for acquired XDR TB was similar (8.9%), that for acquired resistance to fluoroquinolones was slightly higher (11.4% vs. 9.1%), and that for an injectable drug was lower (7.8% vs. 10.6%). In PETTS, rates for acquired XDR TB in GLC-approved countries ranged from 0.6% to 9.8% compared with 6.3% to 18.0% for non–GLC-approved countries (*11*). On the basis of these findings, Georgia is on the higher end of acquired resistance rates for GLC-approved countries. Another recent study of patients in the United States found that among MDR TB patients, the rate of acquired resistance was 6.4% for fluoroquinolones and 6.6% for injectable drugs (*7*). These findings are probably overestimates because only patients with an initial and follow-up DST were included (<30% of all MDR TB patients during the study). Our results, along with the above findings, indicate that acquired resistance occurs at fairly high rates across diverse settings and stress the need for repeating DST among patients with persistent positive sputum cultures. As DST and molecular drug-resistance testing become more available, we will probably see additional reports of acquired resistance and its effects.

Our results provide novel data on risk factors for acquired resistance among MDR TB patients and indicate that severity of disease at baseline and persistent AFB smear positivity were predictors of acquired resistance. Patients with a higher AFB smear microscopy grade (indicating higher bacillary load), baseline cavitary disease, and increasing baseline drug resistance had higher rates of acquired resistance. Persons with a lower baseline body mass index tended to have higher acquired resistance, but this result was not significant (p = 0.053). Shin et al. also found that baseline cavitary disease was associated with acquired XDR TB (aOR, 3.47, 95% CI 1.32–9.14), and although baseline drug resistance was not modeled, they found that a history of treatment with an injectable drug was a risk factor for acquired XDR TB (*12*). PETTS results corroborate our findings of increasing baseline drug resistance leading to higher rates of acquired resistance. Although that study reported that cavitary disease was associated with acquired XDR TB by univariate analysis (relative risk 1.84, 95% CI 1.04–3.26), it was included only as part of a propensity score for multivariate analysis and not modeled separately (*11*).

A novel finding of our study was the association of persistent smear positivity at 4 and 6 months with acquired resistance. Because AFB smear testing is more widely available than culture, this is a practical test that can help clinicians target high-risk patients who might need a regimen change, improved adherence, or other intervention.

We previously demonstrated acquired resistance among *M. tuberculosis* isolates from resected cavitary tissue compared with sputum samples (*13*). The cavitary lesion is an ideal setting for acquired resistance, given high bacterial loads, active mycobacterial replication, reduced exposure to host defenses, and potentially low penetration by drugs. The fibrotic wall of the cavity and variable vascularization might decrease SLD drug penetration, result in drug-selection pressure, and lead to emergence of acquired resistance (*22*). We are currently conducting a pharmacologic study to measure cavitary penetration of SLDs to assess the association between drug penetration and acquired resistance. It has been shown in an in vitro system that pharmacokinetic variability can lead to emergence of MDR TB (*23*). Consistent with this finding is a study that showed that among drug-susceptible TB patients, low isoniazid and rifampin concentrations preceded all cases of drug resistance (*24*). However, no clinical studies of SLD pharmacokinetics have examined their relationship with acquired resistance and treatment outcomes.

High rates of poor outcomes among MDR TB patients with isolates that have acquired resistance in our cohort are a concern and stress the need for prevention of acquired resistance. Only 2 patients with isolates that had acquired resistance had favorable outcomes, 1 who had adjunctive surgery and 1 whose isolate remained susceptible to ofloxacin, capreomycin, and kanamycin. An increasing number of reports have found that adjunctive surgery might be beneficial for MDR TB patients with cavitary disease (*25*,*26*). However, these studies were observational, and a randomized controlled clinical trial is needed to demonstrate if adjunctive surgery improves MDR TB treatment outcomes, including among patients with isolates that had acquired resistance.

The few other studies reporting some association of acquired resistance and outcomes found results mirroring our findings; however, our study found that acquired resistance associated with a negative outcome in adjusted analysis when controlling for other potential confounders. In a study from Abkhazia, all patients with isolates that had acquired resistance to ofloxacin had a poor outcome (*21*). In a report of 87 MDR TB patients from Uzbekistan, only 5 (28%) of 18 patients with isolates that had acquired resistance to ofloxacin were successfully treated (*27*). In the study by Shin et al., only 14.7% of MDR TB patients in whom XDR TB developed were cured or completed treatment compared with 68.5% among those in whom XDR TB did not develop (*12*).

A limitation of our study was lack of genotyping, which prevented distinguishing isolates that had acquired resistance from reinfection with another strain. Other reports found that potential reinfection with an exogenous strain accounted for 0%–34% of acquired drug resistance (*8*,*11*,*21*,*27*). It has also been estimated that certain strains of *M. tuberculosis* have higher mutation rates and are more likely to acquire drug resistance (*28*). In addition, we did not have detailed information on treatment adherence, which prevented us from measuring the association of different levels of adherence with isolates that had acquired resistance. Shin et al. found that cumulative months with <80% treatment adherence were associated with acquired resistance (*12*). The high rate of LFU also prevented determining the association of isolates that had acquired resistance with failure and death in these patients. In addition, lack of DST for many patients who were culture positive at 4 and 6 months might have led to an underestimation of isolates that had acquired resistance and biased the association of isolates that had acquired resistance to ofloxacin and poor outcomes if DST was selectively performed for sicker patients.

In summary, our results provide novel findings on risk factors for *M. tuberculosis* isolates developing acquired resistance and complete analysis of isolates that had acquired resistance and treatment outcomes among MDR TB patients. The need is urgent to further elucidate mechanisms of acquired resistance among *M. tuberculosis* isolates to improve treatment outcomes among MDR TB patients and to ensure that we preserve the effectiveness of newly introduced TB drugs.
